# Distinct NG2 proteoglycan-dependent roles of resident microglia and bone marrow-derived macrophages during myelin damage and repair

**DOI:** 10.1371/journal.pone.0187530

**Published:** 2017-11-02

**Authors:** Karolina Kucharova, William B. Stallcup

**Affiliations:** Cancer Center, Tumor Microenvironment and Cancer Immunology Program, Sanford Burnham Prebys Medical Discovery Institute, La Jolla, California, United States of America; University Medical Center of the Johannes Gutenberg University of Mainz, GERMANY

## Abstract

We used a bone marrow transplantation approach to distinguish the activities of bone marrow-derived macrophages from the activities of central nervous system-resident microglia in phenomena associated with axon demyelination and remyelination. We transplanted wild type or germline NG2 null beta-actin-EGFP expressing bone marrow into irradiated wild type or NG2 null recipient mice, followed by analysis of lysolecithin-induced spinal cord demyelination and remyelination and quantification of Iba-1^+^/ F4/80^+^/ EGFP^+^ macrophages and Iba-1^+^/ F4/80^+^/ EGFP^-^ microglia. One week after microinjection of 1% lysolecithin into the spinal cord, wild type recipients receiving NG2 null bone marrow exhibit greatly reduced infiltration of macrophages into lesions, compared to wild type recipients receiving wild type bone marrow. Wild type bone marrow recipients also exhibit larger numbers of demyelinated axons than NG2 null recipients, indicative of macrophage participation in the initial myelin damage. However, wild type bone marrow recipients also exhibit superior myelin repair at 6 weeks post-injury, compared to NG2 null bone marrow recipients, demonstrating the additional importance of macrophages in remyelination. Incompletely repaired lesions in NG2 null bone marrow recipients at 6 weeks post-injury retain elevated numbers of macrophages, in contrast to lower numbers of macrophages in more completely repaired lesions in wild type bone marrow recipients. This suggests that NG2 expression renders macrophages more effective in myelin repair and less likely to promote chronic inflammation. Effective macrophage involvement in myelin repair is due in part to effects on the proliferation and/or recruitment of oligodendrocyte progenitor cells. Reduced numbers of oligodendrocyte progenitors are seen in lesions in NG2 null bone marrow recipients, likely due to deficits in macrophage production of oligodendrocyte progenitor-relevant mitogens and in phagocytosis of inhibitory myelin debris. Microglia also appear to be important for clearance of myelin debris, as indicated by reduced phagocytosis in NG2 null recipients receiving wild type bone marrow.

## Introduction

The pathology of multiple sclerosis (MS) is complex due at least in part to the participation of several different cell types in disease progression [1–6]. In addition to oligodendrocytes and neurons (axons), immune cells and other elements of the microenvironment, such as blood vessels, play prominent roles in both myelin damage and repair. Defining the respective contributions of these various cell types will be required for a more complete understanding of MS and for improving treatment of MS patients. We have previously shown that the NG2 proteoglycan is expressed by three different populations of cells in demyelinated lesions induced in mouse spinal cord by microinjection of lysolecithin [7, 8]. Oligodendrocyte progenitor cells (OPCs), pericytes, and myeloid cells (macrophages and microglia) all contribute to NG2 expression in these lesions. Using Cre-Lox technology, we have specifically ablated NG2 in two of these populations, OPCs and myeloid cells [8]. This has led not only to initial understanding of NG2 function in these cell types, but also to the generation of mouse models characterized by reduced participation of OPCs and myeloid cells in lesion formation and repair.

OPC-specific ablation of NG2 was previously accomplished by crossing NG2 floxed mice with Olig2-Cre partners [8]. Use of OPC-specific NG2 null (OPC-NG2ko) mice revealed an important role for NG2 in OPC proliferation, leading to generation of reduced numbers of myelinating oligodendrocytes and to diminished myelin repair. Myeloid-specific ablation of NG2 using crosses between NG2 floxed mice and LysM-Cre mice allowed us to define NG2-dependent roles of myeloid cells in both myelin damage and repair [8]. In myeloid-specific NG2 null (My-NG2ko) mice, recruitment of myeloid cells to demyelinated lesions was greatly reduced. This appears to be a general consequence of NG2 ablation, since reduced myeloid recruitment is also observed in brain tumor progression in My-NG2ko mice [9–11]. The myeloid cell deficit in the demyelination model was accompanied by the generation of much smaller demyelinated lesions than in control mice, demonstrating a role for macrophages in the process of myelin damage. In spite of these smaller lesions, the process of myelin repair was also impaired in My-NG2ko mice, as evidenced by larger numbers of demyelinated axons at 6 weeks post-injury than seen in control mice. These results echoed a theme set forth in several previous publications; namely, that macrophages can be agents of both damage and repair in the central nervous system [3, 4, 12].

Two shortcomings of the conditional NG2 ablation study [8] were that (a) we were not able to determine clearly the efficiency of NG2 ablation in myeloid cells due largely to the transient nature of NG2 expression in these cells, and (b) we were not able to distinguish between NG2-dependent functions of resident microglia and bone marrow-derived macrophages (BM-Mac), since germline LysM-Cre mediated ablation of NG2 occurs in both cell types. As a means of both confirming and extending the previous findings, we have now used a bone marrow (BM) transplantation approach for generating mice with wild type (WT) and NG2 null (KO) myeloid cells. By transplanting WT and germline NG2 KO recipients with bone marrow from wild type and germline NG2 null β-actin EGFP donors (WTBM and KOBM), we have in fact generated three different lines of chimeric mice (Table 1).

**Table 1:** Chimeric mice from bone marrow transplantation.

Host	BM donor	BM-Mac	Microglia	Pericytes and OPCs	Line designation	EGFP^+^ WBC (%)	EGFP^+^ CD18^+^ (%)
WT	EGFP+ WT	NG2 +	NG2 +	NG2 +	WT-WTBM	97.46 ± 3.7	94.69 ± 3.5
KO	EGFP+ WT	NG2 +	NG2 -	NG2 -	KO-WTBM	95.57 ± 4.6	92.43 ± 2.4
WT	EGFP+ KO	NG2 -	NG2 +	NG2 +	WT-KOBM	95.99 ± 3.5	94.14 ± 2.9

Transplantation of EGFP bone marrow (BM) from wild type (WTBM) and NG2 null (KOBM) donors into irradiated wild type (WT) and NG2 null (KO) recipients was used to generate 3 lines of chimeric mice. The efficiency of bone marrow engraftment was determined as described in Materials and Methods (Bone Marrow Transplantation). Values shown here represent the percentages of white blood cells (WBC) that are EGFP-positive in each of the chimeric lines. Of importance for the current work, similar EGFP engraftment efficiency is seen for circulating myeloid cells using the β2 integrin marker CD18.

WT-WTBM mice represent controls with a normal distribution of NG2 expression. WT-KOBM mice represent BM-Mac-specific NG2 null mice that retain NG2 expression in microglia, OPCs, and pericytes. KO-WTBM mice represent mice that retain NG2 expression in BM-Mac, but lack NG2 in OPCs, pericytes, and microglia. These mice have allowed us to study the importance of NG2 in a system that is independent of LysM-Cre mediated NG2 ablation. As a result of germline NG2 ablation, we are now assured that NG2 is completely ablated in either BM-Mac (WT-KOBM) or in resident microglia (KO-WTBM). Moreover, these studies have allowed us to distinguish the activities of macrophages and microglia based on EGFP expression by the macrophages. We have chosen not to study KO-KOBM mice in this report because the complete absence of NG2 in both host and donor cells does not specifically enhance our understanding of myeloid cells to the same extent as results obtained in WT-KOBM and KO-WTBM mice. Excluding KO-KOBM mice also simplifies presentation of the results in the various figures and tables.

Results from these studies, based on bone marrow transplantation, confirm many of our previous Cre-Lox based observations concerning the importance of NG2 during axon demyelination and remyelination. In addition, we now have a better understanding of the respective NG2-dependent roles of macrophage and microglial populations during myelin damage and repair.

## Materials and methods

### Animals

Animal work was performed in the vivarium of the Sanford Burnham Prebys Medical Discovery Institute in strict accordance with the recommendations set forth in the National Institutes of Health Guide for the Care and Use of Laboratory Animals. Our vivarium is fully accredited by the American Association for the Accreditation of Laboratory Animal Care (AAALAC). All experimental protocols were approved by the Institutional Animal Care and Use Committee (IACUC) of the Sanford Burnham Prebys Medical Discovery Institute (animal usage protocol 15–107). For bone marrow transplantation (via retro-orbital injection), mice were anesthetized with Isoflurane. For lysolecithin injection, mice were anesthetized using Ketamine/Xylazine. For terminal transcardial perfusion, mice were also anesthetized with Ketamine/Xylazine. All efforts were made to minimize animal discomfort and suffering.

### Bone marrow transplantation

C57BL/6 wild type and germline NG2 null mice on a β-actin-EGFP background were used as donors for bone marrow (BM) transplantation, as previously described [8, 13, 14]. Germline NG2 null mice have a neo cassette inserted into the third NG2 exon to inactivate expression of the proteoglycan [15]. Gamma-irradiated [a split dose totaling 10 Gy] wild type and germline NG2 null mice served as recipients for EGFP-positive wild type and germline NG2 null bone marrow. Bone marrow was harvested from euthanized wild type and germline NG2 null β-actin EGFP donor mice. Dissected femurs and tibiae were flushed with sterile 0.1 M PBS containing 5 mM EDTA and 2% FCS, and red blood cells were lysed by addition of two volumes of ACK buffer. Surviving bone marrow cells were washed, filtered through a nylon mesh, and resuspended in sterile 0.1 M PBS containing 2% mouse serum. Bone marrow cells were not further purified prior to transplantation. Recipient mice each received 700,000 bone marrow cells via retro-orbital injection. Chimeric mice were maintained on antibiotic water [neomycin sulfate, 1.1 g/l, and polymyxin B sulfate, 455 mg/l] until used for experimentation. After a 6-week recovery, peripheral blood was drawn, subjected to lysis with ACK buffer, and analyzed by flow cytometry for the percentage of all white blood cells (WBC) that were positive for EGFP. At least 10,000 blood cells from each mouse were analyzed to establish the extent of EGFP bone marrow engraftment. WBC from EGFP-positive wild type mice were used as controls for this analysis, serving to define the 100% engraftment level. The efficiency of engraftment in WT-WTBM, KO-WTBM, and WT-KOBM mice was established by comparison to this 100% standard (see Table 1). Flow cytometric determination of EGFP-positive, CD18-positive cells further served to establish the engraftment efficiency for myeloid cells (Table 1).

### Lysolecithin-induced spinal cord demyelination

Spinal cord demyelination via microinjection of lysolecithin was carried out as previously described [7, 8], using mice anesthetized with ketamine/xylazine [100/10 mg/kg] administered intraperitoneally. A 1.5 μl solution of 1% lysophosphatidylcholine [L-α-lysolecithin; Sigma L1381] in 0.1 M PBS was microinjected at a rate of 0.5 μl/min into the white matter between the Th12 and Th13 vertebrae, just lateral to the posterior spinal vein, at depths of 0.8 and 0.4 mm [i.e. 1.5 min at each depth]. The needle was left in place for an additional 2 min to avoid backflow. Sham-operated controls consisted of identical injections with 0.1M PBS. For final analyses, animals were deeply anesthetized with ketamine/xylazine, and transcardially perfused with 0.1 M PBS, followed by 4% paraformaldehyde (pH 7.4).

Four mice were analyzed for each of the 5 parameters studied in the Results and Discussion section [NG2 expression, myeloid cell abundance in demyelinated lesions, phagocytosis of myelin debris, OPC abundance in demyelinated lesions, and extent of axon demyelination and remyelination]. A replicate set of experiments was also performed with independent cohorts of mice to insure the reproducibility of the reported phenomena. Outcomes from the two sets of experiments were very similar, allowing us to pool the data from both sets to generate the results presented in this report.

### Tissue preparation and immunohistochemistry

2000 μm spinal cord segments were sectioned from each spinal cord, representing 1000 μm on each side of the injection site. 5 sections were selected from each spinal cord at intervals of 500 μm in order to span the entire 2000 μm segment. These sections were immunolabeled as previously described [7, 8]. The following primary antibodies were used: 1) guinea pig or rabbit anti-NG2 (1:50 or 1:200; [16]; 2) rabbit or rat anti-PDGFRα [17] or eBioscience, 1:200); 3) rabbit antibody against PDGFRβ [16], 4) mouse, rat or rabbit anti-myelin basic protein (MBP, Sternberger, clone SMI-94, Invitrogene or Origene, 1:500); 5) rat anti-CD18 (eBioscience, 1:200); 6) rabbit anti-IBA1 (Wako, 1:1000; 7) mouse anti-Pan-Axonal Neurofilament (smi-312R, Sternberger, 1:1000); 8) rabbit anti-Olig2 (Abcam or Phosphosolution, 1:200). Highly cross-adsorbed donkey secondary antibodies conjugated to Alexa488, CY3 and/or Alexa 647 were obtained from Jackson ImmunoResearch and diluted 1:250. 4’-6-diamidino-2-phenylindole (DAPI, 4 μg/mL, D3571, Invitrogen) was used for general nuclear staining of all sections. Labeled sections were mounted on slides, air-dried, and then cover-slipped with Vectashield (H-1000, Vector lab).

### Image processing and quantification

At least 4 WT-WTBM, 4 KO-WTBM, and 4 WT-KOBM mice were examined for quantification of Iba-1^+^/ F4/80^+^ EGFP^+^ macrophages, Iba-1^+^/ F4/80^+^ EGFP^-^ microglia, and PDGFRα^+^/Olig2^+^ OPCs at 1 and 6 weeks after microinjection of lysolecithin. Sham-operated mice injected with 1.5 μL of 0.1M PBS were used as controls for comparisons with all lysolecithin-injected mice.

Anti-NG2 antibody in conjunction with markers for macrophages, microglial cells, and non-myeloid cells (including both OPCs and pericytes) was used to quantify NG2 expression in these cell types. Image Pro Plus 5.1 software was used to quantify total NG2 pixel density associated with the respective immunolabeled cell types.

Quantitative analysis of phagocytosed myelin debris was performed via double staining with antibodies against MBP and Iba-1. Sections were scanned via confocal microscopy (LSM 710 NLO Zeiss; ZEN 2010), and colocalization of MBP-positive myelin debris in Iba-1 positive myeloid cells was evaluated using Image-Pro Plus 5.1 (Media Cybernetics).

In order to identify non-phagocytized MBP (i.e. intact myelin), phagocytized MBP pixels associated with Iba-1-positive cells were subtracted from total MBP pixels. For quantifying the extent of axon myelination in WT-WTBM, WT-KOBM, and KO-WTBM mice at 1 and 6 weeks after lysolecithin microinjection, sections were double-labeled with pan-neurofilament (NF) and MBP antibodies. The extent of MBP-NF association were quantified by confocal microscopy using Image-Pro Plus software.

In all cases of pixel density quantification, the intensity threshold representing positively labeled areas was chosen approximately 30% above the background level of labeling, as established by multiple observations with the spinal cord tissues under examination.

### Statistical analysis

Data were analyzed for statistical significance using un-paired t-tests and ANOVA. P-values less than 0.05 were considered statistically significant.

## Results and discussion

Immediately following gamma irradiation of recipient mice, bone marrow transplantations were carried out using β-actin-EGFP donors as described in Materials and Methods. As described below, after a 6-week recovery period, these mice were used for lysolecithin microinjection and subsequently for several types of analyses designed to elucidate the role of NG2 in macrophage and microglial contributions to myelin damage and repair.

### NG2 expression

Expression of NG2 by various host and donor cell types and the effectiveness of NG2 ablation were assessed by immunolabeling for NG2 in conjunction with the EGFP marker. Previous studies have shown that only 5% of bone marrow cells are positive for NG2, and that only 2% of bone marrow cells are hematopoietic in nature as determined by co-expression of both NG2 and CD34 [14]. Moreover, additional studies suggest that NG2 is not expressed to any large extent by circulating monocytes. Myeloid expression of NG2 appears to occur only after activation of these cells in response to inflammatory signals [9, 11]. This observation is in line with several additional reports of transient NG2 expression in myeloid cells following CNS injury [17–22]. A recent failure to observe myeloid expression of the NG2 gene in an EAE model [23] may be attributable to the transient nature of NG2 expression, as noted by the authors of this report.

**Fig 1:**
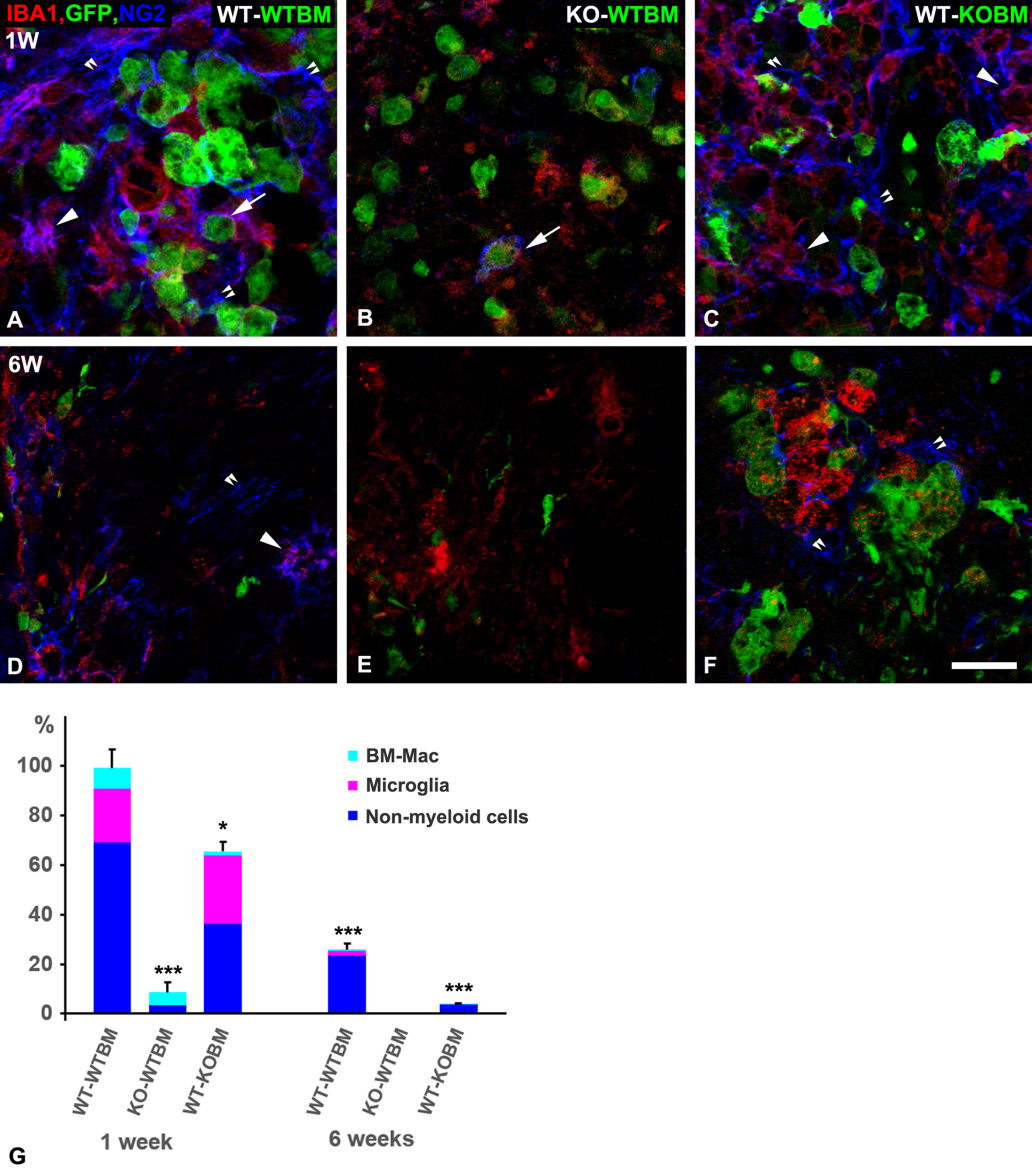
NG2 expression 1 and 6 weeks after demyelination. Image Pro Plus 5.1 software was used to quantify NG2 pixels associated with three classes of cells: macrophages, microglial cells, and non-myeloid cells (this includes both OPCs and pericytes). These cell types were identified by immunolabeling according to the following strategy. 1-week after demyelination in wild type (WT) recipient mice that received WT bone marrow (WT-WTBM), the majority of NG2 expression (blue) occurs in non-myeloid OPCs and pericytes (double arrowheads). NG2 (blue) is also expressed by some IBA1 (red) positive, EGFP-positive macrophages (green; arrow) and by some IBA1 positive, EGFP-negative microglia (arrowhead). (B) In contrast, NG2 expression is greatly reduced in lesion sites in NG2 null recipients that received wild type bone marrow (KO-WTBM). (C) In wild type recipients that received NG2 null bone marrow (WT-KOBM), NG2 is seen in non-myeloid cells (double arrowheads) and in microglia (arrowhead), but not in EGFP-positive macrophages. (D) 6-weeks after demyelination in WT-WTBM mice, overall NG2 expression is only about 30% of that seen at 1-week (G) and is seen mainly in OPCs (double arrowhead). NG2 is occasionally seen in IBA1-positive microglia (arrowhead). (E) In KO-WTBM mice at 6-weeks, NG2 is almost completely absent. (F) In WT-KOBM mice at 6-weeks, the small residual NG2 expression is by non-myeloid cells (double arrowheads). NG2 quantification is summarized in panel G, where total NG2 pixel density in WT-WTBM at 1 week after demyelination is defined as 100%. All other values are compared to this standard. Scale bar indicates 30 μm (A-F). * p < 0.05; *** p < 0.001.

Expression of EGFP and the myeloid marker Iba-1 were used to distinguish bone marrow-derived macrophages from CNS resident microglia (Fig 1). Bone marrow-derived macrophages are identified as EGFP-positive, Iba-1 positive cells, while resident microglia are identified as EGFP negative, Iba-1 positive cells. The overall NG2 pixel density in demyelinated lesions in WT-WTBM mice at 1-week post-lysolecithin injection is defined as the 100% expression level. Based on our previous experiments, this level of NG2 expression is more than 10 times greater than that observed in sham operated animals, demonstrating the up-regulation of NG2 expression in response to the demyelinating injury. Almost 70% of this NG2 is contributed by host oligodendrocyte progenitor cells (OPCs) and pericytes, while about 20% and 10% are contributed by microglia and bone marrow-derived macrophages, respectively. NG2 expression in lesions in KO-WTBM mice is less than 10% of that seen in WT-WTBM mice, owing to the ablation of NG2 expression in host OPCs, pericytes, and microglia. Derivation of a small number of pericytes from the bone marrow accounts for a few percent of this NG2, while bone marrow-derived macrophages also contribute a small percentage. This macrophage NG2 is almost completely absent in lesions in WT-KOBM mice, while microglial NG2 and OPC/pericyte NG2 levels remain substantial. The decrease in non-myeloid NG2, compared to WT-WTBM mice, is likely due primarily to reduced numbers of OPCs (see section on “OPC abundance in demyelinated lesions”) in the case of lesions with decreased macrophage infiltration. This is a phenomenon that we have noted previously [8], and which will be discussed further in a later section on OPC abundance. At 6 weeks post-injury, this OPC contribution to NG2 expression in WT-WTBM and WT-KOBM mice is further reduced by differentiation of OPCs to mature oligodendrocytes.

### Myeloid cell abundance in demyelinated lesions

Our previous work established that NG2 ablation resulted in greatly reduced numbers of myeloid cells in demyelinated lesions [7–9]. However, these studies did not distinguish between the effects of NG2 ablation on bone marrow-derived macrophages versus resident microglia. In the current study, immunolabeling for the macrophage markers Iba-1 and F4/80 in conjunction with the EGFP label associated with donor bone marrow allows us to make this distinction between macrophages and microglia. We used this strategy to quantify macrophages and microglia in 1-week and 6-week lesions in the three lines of mice. EGFP-positive cells are not observed in the spinal cords of sham-operated mice that did not receive lysolecithin injection. Recruitment of EGFP-positive myeloid cells from the circulation therefore only occurs in response to the lysolecithin insult.

**Fig 2:**
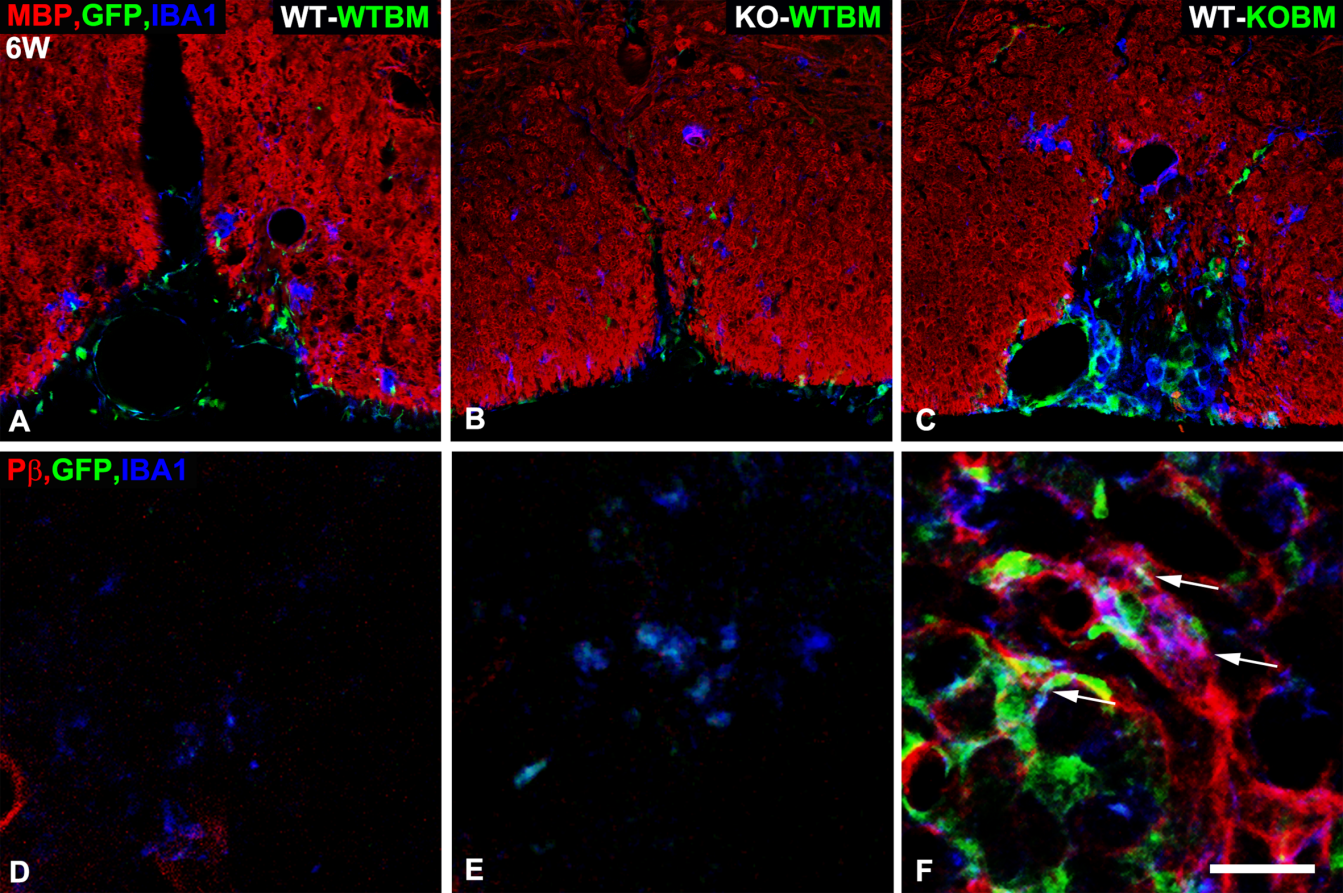
Persistence of atypical NG2 null macrophages in lesions in WT-KOBM mice. Sections from lesions at 6-weeks after lysolecithin injection were evaluated for myeloid cell abundance by use of the EGFP marker (green) and immunolabeling for Iba-1 (blue). **A-C**. Immunolabeling for MBP (red) allows visualization of the extent of myelin repair. A. WT-WTBM. B. KO-WTBM. C. WT-KOBM. IBa-1-positive, EGFP-positive macrophages remain prominent in areas of the WT-KOBM spinal cord in which myelin repair is incomplete. **D-F**. Immunolabeling for Iba-1 and PDGFRβ (red) in conjunction with the EGFP marker identifies atypical macrophages that express PDGFRβ. D. WT-WTBM. E. KO-WTBM. F. WT-KOBM. Many persistent macrophages in lesions in WT-KOBM mice co-express Iba-1, EGFP, and PDGFRβ (arrows). Scale bar = 100 μm (A-C) and 25 μm (D-F).

The EGFP/Iba-1 labeling methodology reveals that lesions in WT-WTBM mice contain 111 EGFP^+^Iba-1^+^ macrophages per 0.1 mm^2^ area at 1-week after lysolecithin microinjection (Table 2). This value drops to a low level of only 6 macrophages per 0.1 mm^2^ after an additional 5 weeks of recovery. Lesions in KO-WTBM mice exhibit somewhat lower numbers of macrophages at 1-week (73 per 0.1 mm^2^), a value that drops to 8 macrophages at the 6-week time point. In contrast, 1-week lesions in WT-KOBM mice contain only 22 macrophages per 0.1 mm^2^, confirming our previous finding that NG2 ablation greatly reduces initial macrophage infiltration into lesions [8]. Strikingly, 18 macrophages per 0.1 mm^2^ are still found in WT-KOBM lesions at 6-weeks post-injury. Moreover, about 30% of these persistent NG2 null macrophages express PDGFRβ, a marker not seen on NG2-positive macrophages in lesions in either WT-WTBM or KO-WTBM mice 6 weeks after demyelination (Fig 2). Interestingly, activation of PDGFRβ has been linked to maintenance of macrophages in a less differentiated state [24, 25]. It is thus tempting to conclude that there is a connection between the poor myelin repair observed in WT-KOBM mice (see Fig 2C and the section on “Extent of axon demyelination and remyelination”) and the persistence of these undifferentiated NG2 null macrophages in the lesion (Fig 2C and 2F).

**Table 2:** Abundance and phagocytic activity of myeloid cells in demyelinated lesions.

	1 week		6 weeks	
	Cells in 0.1mm^2^	% cells with phagocytosed MBP	Cells in 0.1mm^2^	PDGFRβ ^+^
				Cells in 0.1mm^2^
**WT-WTBM**				
Iba-1^+^/ F4/80^+^/ EGFP^+^ Mac	111 ± 14.4	27 ± 3%	6.29 ± 1.5	0 ± 0
Iba-1^+^/ F4/80^+^/ Microglia	11.63 ± 2	45 ± 7%	3.26 ± 0.9	0 ± 0
**KO-WTBM**				
Iba-1^+^/ F4/80^+^/ EGFP^+^ Mac	72.9 ± 12.6*	36 ± 3%*	7.67 ± 1.3	0 ± 0
Iba-1^+^/ F4/80^+^/ Microglia	11.34 ± 1.8	25 ± 6%*	3.95 ± 1.2	1.46 ± 0.3***
**WT-KOBM**				
Iba-1^+^/ F4/80^+^/ EGFP^+^ Mac	21.73 ± 2.5***	13 ± 4%**	18 ± 2.9**	5.26 ± 1***
Iba-1^+^/ F4/80^+^/ Microglia	59.64 ± 13***	30 ± 3%*	10.24 ± 1.2**	2.01 ± 0.3***

Numbers of Iba-1^+^/ F4/80^+^/ EGFP^+^ macrophages and Iba-1^+^/ F4/80^+^/ EGFP^-^ microglia were determined per 0.1 mm^2^ area in lesions at 1-week and 6-weeks after demyelination. At 6-weeks, numbers of PDGF-R beta (PDGF-Rβ^+^) expressing macrophages and microglia were also determined. Values represent means ± S.D. Statistically significant differences are indicated by

* < 0.05

** < 0.01

*** < 0.001 for values compared to WT-WTBM macrophages or microglia at the same post-injection week. Additional immunostaining for MBP allowed determination of the percentage of macrophages and microglia that had phagocytosed myelin debris. Percentage of phagocytic macrophages = MBP^+^Iba-1^+^EGFP^+^/ Iba-1^+^EGFP^+^ x 100. Percentage of phagocytic microglia = MBP^+^Iba-1^+^EGFP^-^/Iba-1^+^EGFP^-^ x 100. Essentially identical results were obtained when F4/80 was used as the myeloid cell marker. Because our engraftment efficiency is around 95% in each of the chimeric mouse lines (see Table 1), a few surviving recipient macrophages will be mistakenly scored as EGFP-negative microglia in each of these trials. Microglial abundance is therefore slightly inflated in all cases.

The 35% decrease in macrophage abundance in 1-week lesions in KO-WTBM mice might be explained by NG2-dependent changes in the recipient microenvironment (due to NG2 ablation). Since we did not previously observe a decrease in macrophage numbers in lesions in OPC-NG2ko mice [8], it seems possible that loss of NG2 by microglia and/or pericytes is responsible for the decrease in macrophage numbers in lesions in KO-WTBM mice. Whether this might be due to reduced microglial/pericyte production of factors needed for recruitment of monocytes/macrophages to sites of inflammation remains to be determined.

In contrast to macrophages, EGFP^-^Iba-1^+^ microglia are present in 1-week lesions in WT-WTBM mice at a density of roughly 11 cells per 0.1 mm^2^ area. This value decreases to about 3 microglia per 0.1mm^2^ at 6-weeks post-injury. In lesions in KO-WTBM mice, these 1-week and 6-week values are still about 11 and 3, respectively, suggesting that NG2 ablation in the host has little effect on microglial abundance. Strikingly, microglial abundance in 1-week lesions in WT-KOBM mice rises almost 6-fold to roughly 60 cells per 0.1 mm^2^, dropping to 10 per 0.1 mm^2^ at the 6-week time point. It seems possible that microglial numbers may increase as a means of compensating for the loss of macrophage recruitment in these WT-KOBM mice.

### Phagocytosis of myelin debris

**Fig 3:**
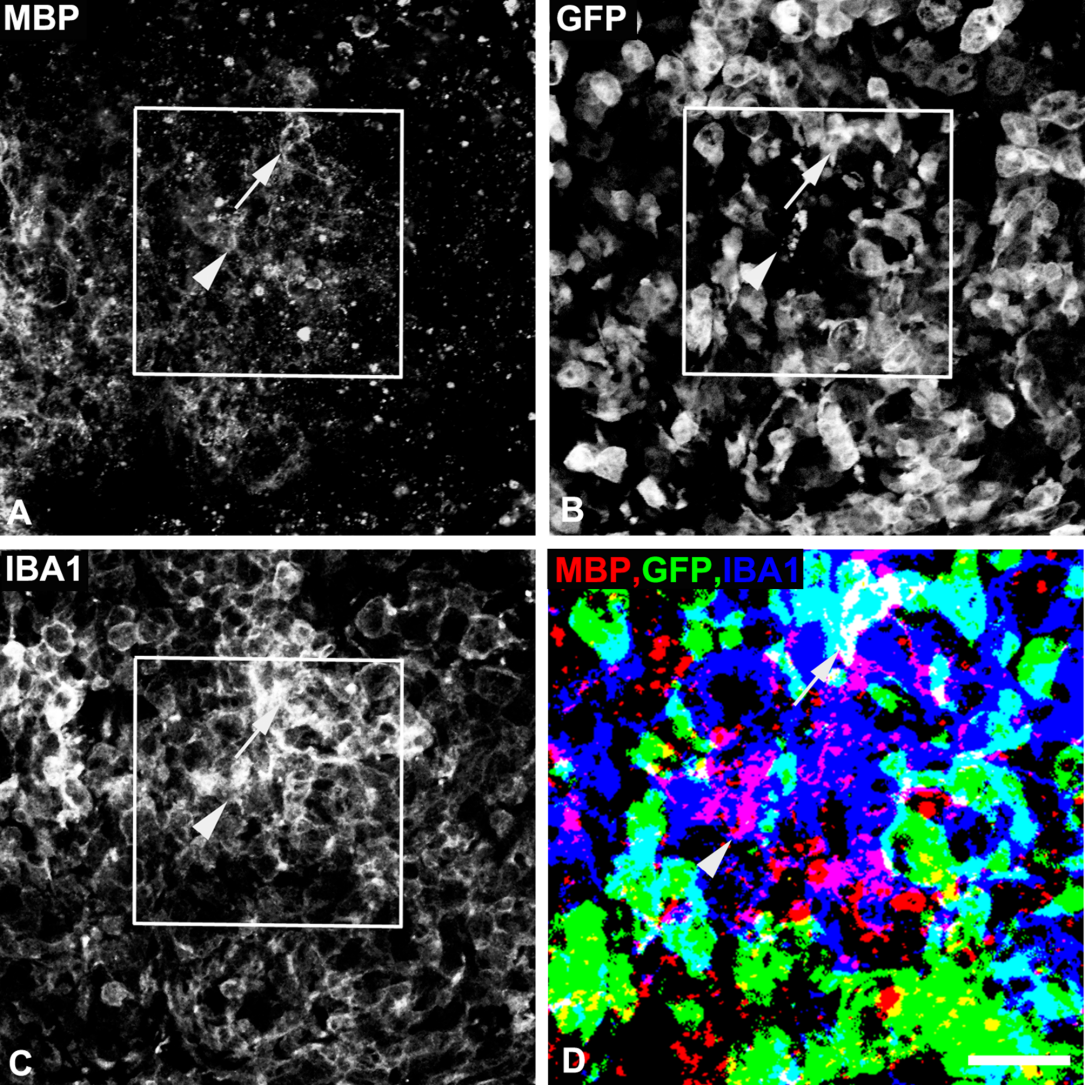
Phagocytosis of myelin debris by macrophages and microglia. Sections from spinal cord lesions one week after lysolecithin microinjection were used to evaluate macrophage and microglial phagocytosis of myelin debris according to the strategy described in Materials and Methods. Cells are examined as being positive for (A) phagocytosed MBP (red), (B) EGFP (green), and (C) Iba-1 (blue). The inset area in panels A-C is shown as a computer-edited merged image at higher magnification in panel D. Phagocytic cells positive for both EGFP and Iba1 are identified as bone marrow-derived macrophages (arrow), while phagocytic cells positive for Iba-1 and negative for EGFP are identified as microglia (arrowhead). In panel D, MBP is readily seen in the Iba1-positive microglial cell (arrowhead). MBP is much less apparent in the Iba1/EGFP-positive macrophage (arrow) due to the strong overlap between EGFP and Iba1 that is seen as white in panel D. Nevertheless, the presence of MBP in both the microglial cell and the macrophage can be seen in panel A, and the Image Pro Plus program allows for MBP quantification in both cell types using merged images (see Table 2). Scale bar = 60 μm in A and 30 μm in B.

Myeloid cell phagocytosis of myelin debris is an important aspect of the myelin repair process. Our previous work suggested that myeloid-specific ablation of NG2 led to impaired phagocytosis of myelin debris [8]), but was not able to attribute this phenomenon specifically to macrophages versus microglia. In the current work, via combined use of the EGFP marker and immunolabeling for MBP and Iba-1, we can evaluate phagocytosis of MBP-positive debris by macrophages and microglia (Fig 3). Table 2 presents these results as the percentage of macrophages that have phagocytosed MBP (Iba-1^+^EGFP^+^MBP^+^/Iba-1^+^EGFP^+^) and the percentage of microglia that have phagocytosed MBP (Iba-1^+^EGFP^-^MBP^+^/Iba-1^+^EGFP^-^) at 1-week post-injury. In lesions in WT-WTBM mice, a higher percentage of microglia appear to be involved in phagocytosis than macrophages. 45% of microglia have phagocytosed myelin debris versus 27% of macrophages. However, the overall impact of microglial phagocytosis may be small compared to macrophage phagocytosis due to the 10-fold higher abundance of macrophages. In lesions in KO-WTBM mice, the percentage of phagocytic microglia decreases to 25%, while the percentage of phagocytic macrophages rises to 36%. In WT-KOBM lesions, phagocytic macrophages decrease to 13% of the population, and phagocytic microglia increase to 30% of the population. The extent of microglial phagocytosis may be especially significant in these mice due to the 6-fold increase in microglial abundance. These observations suggest that NG2 may play a role in the phagocytic function of both macrophages and microglia, since macrophage phagocytic activity declines in WT-KOBM mice while microglial efficiency is reduced in KO-WTBM mice. The failure of microglial activity in WT-KOBM mice to reach the same level seen in WT-WTBM mice could be due to the smaller extent of demyelination in WT-KOBM mice and/or to the 6-fold increase in microglial abundance in WT-KOBM mice; i.e. with more cells, there may be less opportunity for any given cell to phagocytose myelin debris. As noted in the legend to Table 2, our data may slightly inflate microglial abundance, since our 95% engraftment efficiency results in scoring a few EGFP-negative macrophages as microglia.

### OPC abundance in demyelinated lesions

**Fig 4:**
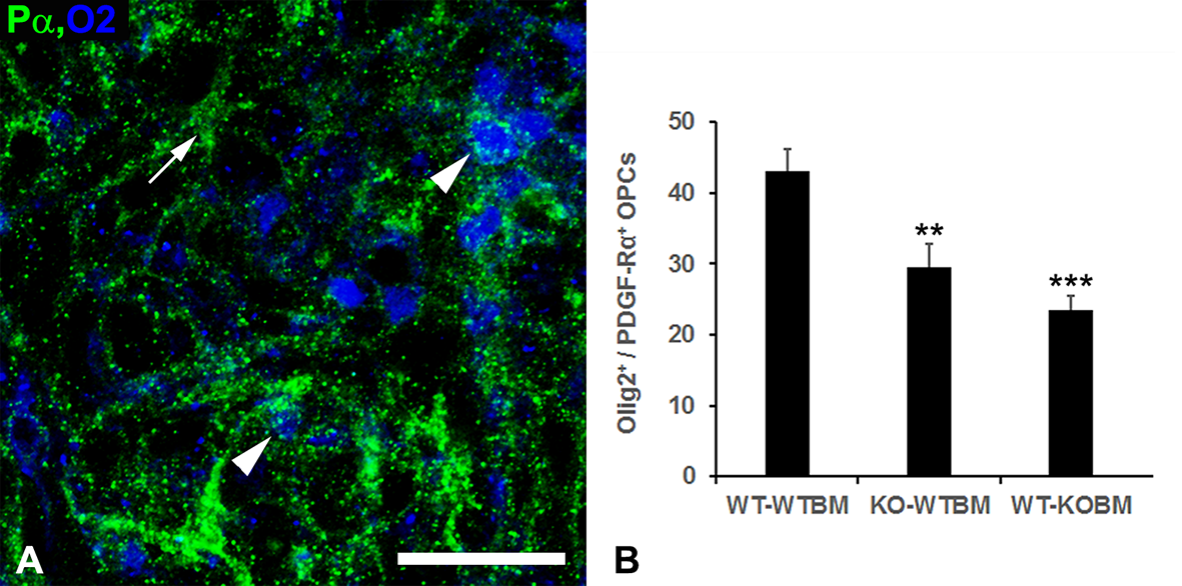
OPC abundance in demyelinated lesions. OPCs in 1-week demyelinated lesions in WT-WTBM mice were identified by double immunolabeling for Olig-2 (blue) and PDGFRα (green). Arrowheads identify OPCs positive for both markers. Arrow identifies a problematic non-OPC cell type that expresses PDGFRα but not Olig2 [8, 17]. These cells are not included in the analysis shown in panel B. Scale bar = 30 μm. (B) Using the labeling scheme illustrated in panel A, OPC numbers are determined per 0.1 mm^2^ area in each of the three chimeric mouse lines. ** p < 0.01; *** p < 0.001.

Our previous work showed that NG2 ablation diminished the abundance of OPCs in lysolecithin-induced demyelinated lesions [7, 8]. This is partly due to the importance of OPC-intrinsic NG2 for the proliferation of these progenitors [26]. However, we also noted a significant stimulatory effect of macrophages on OPC proliferation. When macrophage recruitment was depressed by myeloid-specific ablation of NG2, OPC abundance was decreased in parallel. Macrophages probably exert a dual effect on OPCs due to the phagocytosis of inhibitory myelin debris, as well as to the ability of macrophage-derived factors to promote OPC proliferation. These effects on OPCs are also observed in the current bone marrow transplantation schemes. Compared to 1-week lesions in WT-WTBM mice, lesions in KO-WTBM mice exhibit a 30% reduction in OPC abundance, as defined by double immunostaining for PDGF-Rα and the transcription factor Olig2 (Fig 4). This is most likely a result of ablation of NG2 in the OPCs themselves, but may also reflect the 35% reduction in macrophage number seen in these lesions. Macrophages are known to be an important source of OPC-relevant mitogens [27, 28]. In this same vein, OPC abundance is reduced to an even greater extent (almost 50%) in 1-week lesions in WT-KOBM mice. This decrease is most likely related to the dramatic reduction in macrophage recruitment to these lesions (see Table 2), with significant secondary effects on OPC abundance. These results thus mirror our previous results with OPC-NG2ko and My-NG2ko mice, in which OPC abundance was affected to a greater extent by NG2 ablation in myeloid cells than by NG2 ablation in OPCs [8]. These sets of results are consistent with the concept that loss of macrophage recruitment has a negative effect on OPC abundance. We currently do not have evidence to indicate whether this effect on the OPC population is a direct or indirect consequence of macrophage loss.

### Extent of axon demyelination and remyelination

**Fig 5:**
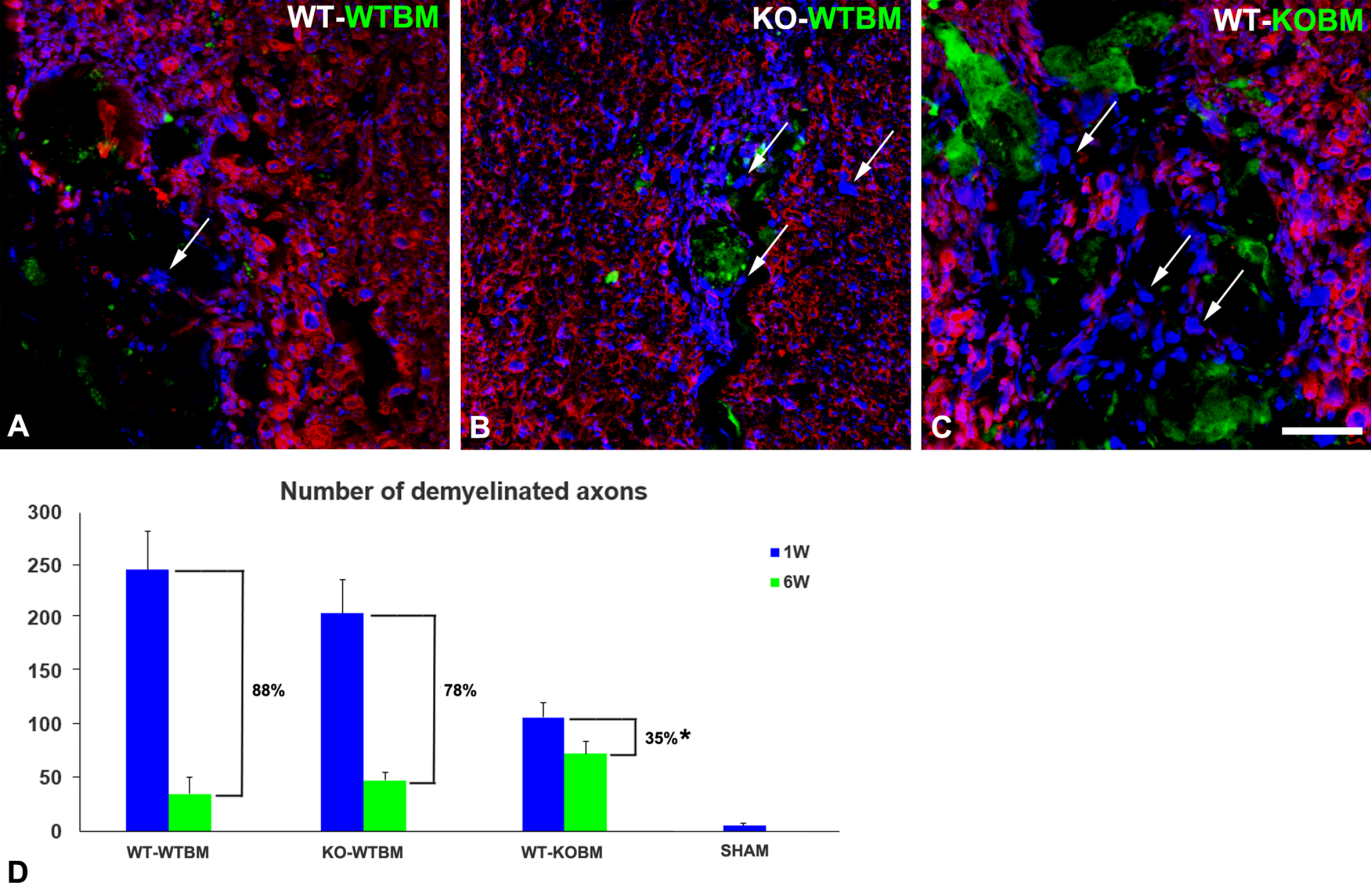
Efficiency of myelin repair at 6 weeks after demyelination event. Double immunostaining for neurofilament protein (NF; blue) and myelin basic protein (MBP; red) was used to quantify numbers of demyelinated axons at 1 week (blue bars in panel D) and at 6 weeks (green bars in panel D) after the demyelinating event. Images in panels A-C are representative of the situation at the 6-week time point. Unmyelinated axons (arrows) are most numerous at the 6-week time point in lesions in WT-KOBM mice. Numbers of unmyelinated axons are determined per 0.1 mm^2^ of lesion area. % remyelination efficiency values (brackets) are determined after subtracting the number of unmyelinated axons (5) seen in sham operated control animals from each of the experimental values. Scale bar = 30 μm. * p < 0.05.

As described previously [8], we have used double immunolabeling for myelin basic protein (MBP) and neurofilament protein (NF) to identify spinal cord axons that are closely associated with myelin (i.e. myelinated axons) versus axons that are not associated with myelin (i.e. unmyelinated axons) (Fig 5). This allows us to quantify the number of unmyelinated axons at 1-week and 6-weeks after lysolecithin microinjection. As a control, sham operated mice exhibit only 5 unmyelinated axons per 0.1 mm^2^ area. In contrast, 250 demyelinated axons per 0.1 mm^2^ are seen in 1-week lysolecithin-induced lesions in WT-WTBM mice, a value that decreases to 30 demyelinated axons after 5 additional weeks of recovery (Fig 5). Taking into account the background value of 5 unmyelinated axons per 0.1 mm^2^ area in sham operated controls, this represents a 88% recovery of axon myelination in lesions in WT-WTBM mice. By comparison, lesions in KO-WTBM mice exhibit 210 demyelinated axons per 0.1 mm^2^ at the 1-week time point, and still have 50 unmyelinated axons at the 6-week time point, representing a 78% recovery of axon myelination. This decrease in myelin repair is largely attributable to the decreased generation of OPCs seen in Fig 4 in lesions in KO-WTBM mice, a set of circumstances we previously noted in lesions in OPC-NG2ko mice [8]. An additional impediment to myelin repair might be the decreased phagocytosis of myelin debris by microglia noted in these lesions (Table 2). Strikingly, lesions in WT-KOBM mice contain only 110 demyelinated axons per 0.1 mm^2^ area 1-week after the demyelination event, but still exhibit 60 demyelinated axons per 0.1 mm^2^ at 6-weeks post-injury, for a repair value only 35%. The reduced number of OPCs in these lesions (Fig 4) is likely to be an important factor in this diminished repair. However, the continued presence of abnormal, PDGFRβ-expressing macrophages in lesions in WT-KOBM mice (Fig 2 and Table 2) may also be a factor in diminished myelin repair in these animals. These findings reinforce the concept that NG2 is required for the role of macrophages in both the initial damage to myelin and subsequent myelin repair. Since CNS resident microglia retain NG2 in both WT-WTBM and WT-KOBM mice, we can attribute these NG2-dependent changes in damage and repair in WT-KOBM mice to the BM-derived macrophages.

## Conclusions

Our previous use of Cre-Lox technology for ablation of NG2 afforded us the ability to assess NG2 function in two specific cell types, OPCs and myeloid cells, during the processes of myelin damage and repair [8]. That approach, however, did not allow us to distinguish between macrophage versus microglial activities, due to LysM-Cre mediated ablation in both cell types, or to determine with certainty how effectively NG2 was ablated in myeloid cells, due to the transience of NG2 expression in those cells. The current bone marrow transplantation approach using germ line NG2 null mice as both donors and recipients assures us that NG2 is completely ablated in either macrophages (in WT-KOBM chimeras) or microglia (in KO-WTBM chimeras). In addition, the expression of EGFP by donor bone marrow cells allows us to distinguish between bone marrow-derived macrophages and resident microglia in order to dissect the respective NG2-dependent contributions of these two cell types.

Our current transplantation-based results support a number of the conclusions previously reached via the Cre-Lox approach [8]. (1) Ablation of NG2 in macrophages (in WT-KOBM chimeras) greatly reduces macrophage recruitment to demyelinated lesions, as previously seen with My-NG2ko mice. (2) Ablation of NG2 in macrophages leads to decreased phagocytosis of myelin debris by macrophages (in WT-KOBM chimeras), confirming the diminished macrophage phagocytosis of myelin debris observed in My-NG2ko mice. Microglia are also active in phagocytosis of myelin debris, a function that is diminished by NG2 ablation (in KO-WTBM chimeras). (3) Ablation of NG2 in OPCs results in decreased numbers of OPCs in demyelinated lesions (in KO-WTBM chimeras), as we previously reported in OPC-NG2ko mice. (4) Ablation of NG2 in macrophages (in WT-KOBM chimeras) results in an even greater decrease in OPC abundance in demyelinated lesions, confirming the effect of macrophage loss on OPC numbers that we previously saw in My-NG2ko mice. The large increase in microglial numbers in WT-KOBM lesions apparently cannot substitute for macrophages in terms of effects on OPCs. (5) Myelin repair is diminished as a result of NG2 ablation in OPCs (in KO-WTBM chimeras), as previously observed in OPC-NG2ko mice. (6) Myelin repair is compromised to an even greater extent when NG2 is ablated in macrophages (in WT-KOBM chimeras), similar to what was seen in My-NG2ko mice.

A new finding of relevance to decreased myelin repair is our observation of a persistent population of PDGFRβ-positive macrophages in the incompletely repaired 6-week lesions in WT-KOBM mice. This population is absent from lesions in WT-WTBM and KO-WTBM chimeras. Since expression of PDGFRβ has been associated with maintenance of macrophages in a more undifferentiated state [24, 25], it is tempting to propose a potential connection between incomplete myelin repair and the continued presence of these aberrant NG2 null macrophages. The involvement of aberrant immune cells in failed myelin repair is very relevant to elucidating the underlying causes of MS, as well as to improving therapy for this disease. Similar to our use of bone marrow transplantation as an investigative tool, depletion of endogenous, flawed immune cells, followed by reconstitution of the immune system using hematopoietic stem cells has been touted as a potentially viable therapy for MS patients [29]. Understanding the properties and activities of immune cells at various stages of demyelination and repair will be essential for effective use of this type of strategy.
